# Prevalence and factors associated with mild depressive and anxiety symptoms in older adults living with HIV from the Kenyan coast

**DOI:** 10.1002/jia2.25977

**Published:** 2022-09-29

**Authors:** Patrick N. Mwangala, Carophine Nasambu, Ryan G. Wagner, Charles R. Newton, Amina Abubakar

**Affiliations:** ^1^ Centre for Geographic Medicine Research Coast Kenya Medical Research Institute (KEMRI) Kilifi Kenya; ^2^ School of Public Health University of the Witwatersrand Parktown South Africa; ^3^ Institute for Human Development Aga Khan University Nairobi Kenya; ^4^ MRC/Wits Rural Public Health and Health Transitions Research Unit (Agincourt) Faculty of Health Sciences, University of the Witwatersrand Johannesburg South Africa; ^5^ Department of Psychiatry University of Oxford Warneford Hospital Oxford UK; ^6^ Department of Public Health Pwani University Kilifi Kenya

**Keywords:** common mental disorders, HIV infection, older adults, prevalence, correlates, Kenya

## Abstract

**Introduction:**

Empirical research on the burden and determinants of common mental disorders (CMDs), especially depression and anxiety, among older adults living with HIV (OALWH) in sub‐Saharan Africa is inadequate. To bridge the gap in Kenya we: (1) determined the prevalence of CMDs among OALWH on routine HIV care compared to HIV‐negative peers; (2) investigated HIV status as an independent predictor of CMDs in older adults; and (3) investigated CMD determinants.

**Methods:**

In a cross‐sectional study conducted between 2020 and 2021, the prevalence of CMDs and associated determinants were investigated at the Kenyan coast among 440 adults aged ≥50 years (257 OALWH). The Patient Health Questionnaire and Generalized Anxiety Disorder scale were administered alongside measures capturing biopsychosocial information. Logistic regression was used to examine the correlates of CMDs.

**Results:**

No significant differences were found in the prevalence of mild depressive symptoms, 23.8% versus 18.2% (*p* = 0.16) and mild anxiety symptoms, 11.7% versus 7.2% (*p* = 0.12) among OALWH compared to HIV‐negative peers, respectively. HIV status was not independently predictive of CMDs. Among OALWH, higher perceived HIV‐related stigma, ageism, increasing household HIV burden, loneliness, increasing functional disability, sleeping difficulties, chronic fatigue and advanced age (>70 years) were associated with elevated CMDs. Among HIV‐negative older adults, loneliness, increased medication burden and sleeping difficulties were associated with elevated depressive symptoms. Easier access to HIV care was the only factor associated with lower CMDs among OALWH.

**Conclusions:**

On the Kenyan coast, the burden of moderate and severe CMDs among older adults is low; however, both OALWH and their HIV‐negative peers have a similar relatively high burden of mild depressive and anxiety symptoms. Our results also suggest that determinants of CMDs among OALWH in this setting are predominantly psychosocial factors. These results highlight the need for psychosocial interventions (at the family, community and clinical levels) to mitigate the risks of mild CMDs as they are known to be potentially debilitating.

## 1 INTRODUCTION

Common mental disorders (CMDs), especially depression and anxiety, are among the leading causes of disability worldwide [1, 2] and cost the global economy about US$1.2 trillion a year [3]. Notably, the global burden of both disorders has not reduced since 1990, despite compelling evidence of cost‐effective interventions [4]. Efforts to address this substantial burden of CMDs should be directed at the most vulnerable in society, including older adults living with HIV (OALWH) residing in sub‐Saharan Africa (SSA). In Kenya, the increasing population of OALWH [5] is experiencing an elevated incidence of chronic age‐related conditions [6], all potentially resulting in an increase of CMDs.

The prevalence of depression among OALWH in SSA ranges from 6% to 59% [7] compared to 26% among their HIV‐negative peers [8]. Outside SSA, estimates of depression among OALWH range from 19% to 45% in Asia [9, 10], 39% to 90% in the United States [11, 12], 16% to 35% in Latin America and the Caribbean [13], and 28% in Europe [14]. Anxiety, on the other hand, ranges from 3% to 21% among OALWH in SSA [7] and 35% to 56% in high‐income countries (HICs) [14, 15].

Understanding the determinants of CMDs among older adults is critical in designing and implementing contextually relevant mental health interventions. A recent review indicated that frequently reported determinants of depression among OALWH were mainly socio‐demographic in nature [7]. Among psychosocial factors, HIV‐related stigma [16], HIV status disclosure [16] and increasing disability scores have been associated with higher odds of depressive symptoms, while social support [17], resilience [16] and spirituality [17] have been shown to be protective factors. Biomedical and lifestyle factors, including alcohol use [17], current/former tobacco smoking [18] and back pain [19], have been associated with elevated levels of depression among OALWH. Among HIV‐negative older adults in SSA, rural residence [20, 21], poor social network [20], living alone [22], being female [8, 20] and a lifetime of unskilled occupation [21] have been associated with elevated odds of depression. No study in SSA has examined the determinants of anxiety among OALWH.

Several reasons call for investigating depression and anxiety among older adults. Firstly, known risk factors for CMD, such as poverty, are more prevalent in older ages [23, 24]. Secondly, CMDs in late life are severely under‐researched and underdiagnosed in primary care [7, 25]. Besides, the prognosis of CMDs among old adults appears to be worse than for young people [26]. Late‐life CMDs may also elevate the risk of developing dementia [27]. Among people living with HIV (PLWH), depression and anxiety have been associated with non‐adherence to combination antiretroviral therapy (cART), risky sexual behaviours, reduced quality of life and higher suicide rates [28, 29, 30]. Given that CMDs are rarely detected but can have serious health impacts on older people, it is increasingly important to assess for mild, moderate and severe levels to determine the scale of the problem [31].

To bridge the gap in Kenya, our study seeks to: (1) determine the prevalence of depressive and anxiety symptoms among OALWH compared to their HIV‐negative peers; (2) investigate HIV status as an independent predictor of depressive and anxiety symptoms in the older adults; and (3) investigate the determinants of CMDs among older adults at the coast of Kenya.

## 2 METHODS

### 2.1 Study design and setting

This cross‐sectional study was conducted at the Kenyan coast in Kilifi and Mombasa Counties between February 2020 and October 2021. The majority of Kilifi residents are rural dwellers whose main form of livelihood is subsistence farming and small‐scale trading [32]. Kilifi has an adult HIV prevalence of 4.5% [33]. Mombasa County borders Kilifi County to the north and is considered urban. The common sources of income in Mombasa county include tourism, wholesale and retail trade. The Mombasa adult HIV prevalence is 7.5% [33].

### 2.2 Study participants and recruitment

#### 2.2.1 Older adults living with HIV

OALWH were recruited from two public HIV specialized clinics in Kilifi and Mombasa Counties. As inclusion criteria, participants had to be aged ≥50 years of age, with a confirmed HIV seropositivity status, and on cART.

In both facilities, we were assisted by community health volunteers or healthcare providers in reviewing existing records to identify all potential participants. We used systematic sampling to identify potential clients for the study.

#### 2.2.2 Older adults without HIV

All HIV‐negative older adults were recruited in Kilifi County. The Kilifi Health and Demographic Surveillance System was used to identify families with eligible older adults. Potential participants aged ≥50 were randomly identified from the existing database and followed up at their homes using GPS coordinates. To be included in the study, individuals had to be ≥50 years, and residents of Kilifi county, and provide consent for participation, including willingness to be tested for HIV using a rapid HIV testing kit (OraQuick) for a confirmation of HIV‐negative status. We chose HIV‐negative adults aged ≥50 years as our comparison group to the OALWH based on previous research [34, 35].

### 2.3 Sample size calculations

Our sample size was calculated using previous studies from Uganda [36] and South Africa [35]. An overall sample size of at least 372 individuals was needed to detect a difference in CMDs between OALWH and HIV‐negative community controls at 80% power and a 5% level of statistical significance. A sample of 450 participants was considered sufficient, allowing for missing data.

### 2.4 Measures

The research instruments were programmed on android tablets using the Research Electronic Data Capture (REDCap) platform [37] for face‐to‐face interviewer administration. All research assistants were trained for 2 weeks by the first author prior to data collection.

#### 2.4.1 Socio‐demographic and asset index form

Socio‐demographic information was captured in REDCap. We also collected information on individual and family ownership of disposable assets for asset index computation. Participants were also asked to provide information on their access to social support, food security in the past week, the number of PLWH within the household and whether they were caring for a sick family member.

#### 2.4.2 General health form

We captured the participant's anthropometric details. Other information included hours spent on sedentary activities in a day, sexual activity, household HIV burden, number of medications one used and common somatic complaints.

Among OALWH, HIV‐specific information questions were asked relating to the disclosure of HIV status and access to HIV care. Information regarding their current cART regimen and overall duration on cART was extracted from medical records. Blood samples were also collected from OALWH for viral load testing.

#### 2.4.3 Psychosocial measures

*The brief 12‐item HIV stigma scale* [38] was utilized to assess participants’ perceived HIV‐related stigma. Higher scores indicate a greater level of perceived stigma. In the current study, this scale yielded good internal consistency alpha, 0.78 (95% CI 0.73, 0.83).

*The 12‐item World Health Organization Disability Assessment Schedule 2 (WHODAS 2)* [39] was used to assess for functional disability among participants. Higher scores indicate a greater level of disability. In this study, the tool demonstrated good internal consistency alpha, 0.77 (95% CI 0.70, 0.84).

*The UCLA 8‐item loneliness scale* [40] was used to assess participants’ perceived loneliness. Higher scores equate to a greater level of loneliness. In the present study, this scale had acceptable internal consistency alpha, 0.61 (95% CI 0.56, 0.64).

*The 20‐item Ageism survey* [41] was used to assess participants’ experiences of ageism. Higher scores indicate more frequent experiences of ageism. In the current study, the internal reliability (Cronbach's alpha) was 0.89 (95% CI 0.87, 0.92).

#### 2.4.4 Measures of common mental disorders

The 7‐item Generalized Anxiety Disorder scale (GAD‐7) [42] and the 9‐item Patient Health Questionnaire (PHQ‐9) [43] were utilized to measure anxiety and depressive symptoms in the previous 2‐week period, respectively. The total scores range from 0 to 21 for GAD‐7 and 0 to 27 for PHQ‐9. For GAD‐7, total scores of 5–9, 10–14 and 15–21 represent mild, moderate and severe anxiety symptoms, respectively [44]. Total scores of 5–9, 10–14 and 15–27 indicate mild, moderate and severe depressive symptoms [45], respectively. A cut‐off score of ≥5 for both PHQ‐9 and GAD‐7 was used to define a positive screen for depressive and anxiety symptoms in the current study, similar to previous studies in Ethiopia [46] and Tanzania [47]. In the present study, the internal consistency alphas for PHQ‐9 and GAD‐7 were good, 0.75 (95% CI 0.70, 0.79) and 0.74 (95% CI 0.70, 0.79), respectively.

#### 2.4.5 Translation of new study measures

All study questionnaires not previously adapted to the local language of Swahili underwent the recommended adaptation procedure in line with international guidelines [48].

### 2.5 Statistical analysis

All analyses were carried out in STATA version 15.0 (StataCorp LP, College Station, TX, USA). Independent Student's *t*‐test and Chi‐square test were used to compare differences in continuous and categorical independent variables, respectively. We used proportions as percentages to estimate the prevalence of CMDs among OALWH and their HIV‐negative counterparts. The Chi‐square test was utilized to compare group differences on binary outcome variables. To investigate HIV status as an independent predictor of CMDs, we utilized logistic regression analyses adjusting for exposure variables that accounted for differences in CMDs. To examine the correlates of CMDs, we used logistic regression models to explore univariate relationships between the binary outcome variables and the different exposure variables. Exposure variables with a *p‐*value < 0.15 in the univariate analysis were subsequently entered into the multivariable models using forward selection (data for OALWH and HIV‐negative older adults were analysed separately for this set of analyses). In all models, collinearity was checked and for all tests of the hypothesis, a two‐tailed *p‐*value < 0.05 was regarded as statistically significant. The overall fit of the final models was examined by Hosmer and Lemeshow (HL) goodness of fit test where a *p‐*value of > 0.05 was considered a good fit. The HL test results were cross‐checked using McFadden's pseudo‐*R* squared statistic.

### 2.6 Ethics approval and consent to participate

The study was approved by the Kenya Medical Research Institute Scientific and Ethics Review Unit (Ref: KEMRI/SERU/CGMR‐C/152/3804). Permission to conduct the study was granted by the research office Kilifi (Ref: HP/KCHS/VOL.X/171) and Mombasa (Ref: COH/Msa/RSC/04). All respondents provided written informed consent for their participation.

## 3 RESULTS

### 3.1 Sample characteristics

A total of 440 participants were included in this study, with a mean age of 60.1 (SD = 6.9) years. The participant response rate was 90%. This included 72 (16%) OALHIV in Mombasa and 368 (84%) in Kilifi. Most participants had formal education (63.2%), were unemployed (65.5%), lived in multigenerational households (81.6%) and cared for a sick family member (66.4%). OALWH were likely to be younger, unmarried, more educated, have lower monthly household income, live alone, with a smaller number of dependents and more food insecure (Table 1).

**Table 1:** Characteristics of the study population by HIV status, *N =* 440

		HIV status		
Characteristic	Total sample *N* = 440	Older adults without HIV, *n* = 183	Older adults living with HIV, *n* = 257	*p*‐value
**Socio‐demographic**				
Age (years)				
50–59	227 (51.6)	84 (45.9)	143 (55.6)	0.02
60–69	171 (38.9)	74 (40.4)	97 (37.7)	
≥70	42 (9.5)	25 (13.7)	17 (6.6)	
Sex				
Female	258 (58.6)	98 (53.6)	160 (62.3)	0.07
Male	182 (41.4)	85 (46.4)	97 (37.7)	
Marital status				
Never married	12 (2.8)	4 (2.2)	8 (3.1)	<0.001^†^
Separated/Divorced/Widowed	181 (41.1)	45 (24.6)	136 (52.9)	
Married/cohabiting	247 (56.1)	134 (73.2)	113 (44.0)	
Education level				
None	162 (36.8)	90 (49.2)	72 (28.0)	<0.001^†^
Primary	182 (41.4)	65 (35.5)	117 (45.5)	
Secondary	73 (16.6)	22 (12.0)	51 (19.9)	
Tertiary	23 (5.2)	6 (3.3)	17 (6.6)	
Employment				
Unemployed	288 (65.5)	126 (68.9)	162 (63.0)	0.1
Employed	116 (26.3)	39 (21.3)	77 (30.0)	
Retired	36 (8.2)	18 (9.8)	18 (7.0)	
Monthly household income (Ksh)				
≤10,000	279 (63.4)	69 (37.7)	210 (81.7)	<0.001
Above 10,000	161 (36.6)	114 (62.3)	47 (18.3)	
Living arrangements				
Multiple generational families	359 (81.6)	169 (92.3)	190 (73.9)	<0.001^†^
Single generational families	41 (9.3)	6 (3.3)	35 (13.6)	
Alone	40 (9.1)	8 (4.4)	32 (12.5)	
Number of dependents, mean (SD)	3.2 (2.6)	3.6 (2.5)	2.9 (2.7)	0.01
Caring for a sick family member, OM = 2				
Yes	291 (66.4)	104 (57.1)	187 (73.1)	0.001
No	147 (33.6)	78 (42.9)	69 (26.9)	
Access to instrumental/social support				
None	199 (45.2)	93 (50.8)	106 (41.2)	0.07
Sometimes	215 (48.9)	83 (45.4)	132 (51.4)	
Most of the time	26 (5.9)	7 (3.8)	19 (7.4)	
Food insecurity (lack of food in the past week), OM = 3				
Never	293 (67.1)	134 (73.6)	159 (62.4)	0.002^†^
Sometimes	119 (27.2)	45 (24.7)	74 (29.0)	
Most of the times/always	25 (5.7)	3 (1.7)	22 (8.6)	
Asset index score^a^—mean (SD)	2.3 (1.5)	1.9 (1.2)	2.5 (1.6)	<0.001
Body mass index—mean (SD), OM = 11	24.9 (6.0)	24.7 (6.1)	25.0 (5.9)	0.7
Loneliness score^b^—mean (SD), OM = 3	13.9 (3.7)	13.0 (3.4)	14.6 (3.7)	<0.001
Functional disability score^c^—mean (SD), OM = 2	2.5 (4.3)	1.5 (3.0)	3.1 (4.9)	<0.001
Ageism score^d^—mean (SD)	4.2 (5.9)	3.0 (4.4)	5.0 (6.6)	<0.001
Hours spent in sedentary activities in a day, mean (SD), OM = 12	4.5 (2.6)	4.3 (2.1)	4.6 (2.9)	0.3
Sexually active, OM = 4				
Yes	206 (47.3)	105 (57.4)	101 (39.9)	<0.001
No	230 (52.7)	78 (42.6)	152 (60.1)	
Sleeping difficulties in the past month, OM = 4				
None	276 (63.3)	125 (68.3)	151 (59.7)	0.01^†^
Sometimes	131 (30.1)	53 (29.0)	78 (30.8)	
Most of the times/always	29 (6.6)	5 (2.7)	24 (9.5)	
Chronic fatigue				
Yes	56 (12.7)	21 (11.5)	35 (13.6)	0.5
No	384 (87.3)	162 (88.5)	222 (86.4)	
Number of medications participants are currently using, mean (SD), OM = 8	1.6 (1.6)	0.4 (1.2)	2.4 (1.2)	<0.001

Note: All numbers are reported as frequencies with percentages unless otherwise stated. *p*‐values are for the difference between OALWH and their HIV‐negative peers by sample characteristic. *p‐*values have been derived from Chi‐square test (or Fisher's exact test) and independent Student's *t*‐test for categorical and continuous independent variables, respectively.

Abbreviations: Ksh, Kenya shillings; OM, observation with missing value; SD, standard deviation.

^a^ Score range = 0–8, higher scores indicate better socio‐economic status.

^b^ Score range = 8–27, higher scores indicate greater loneliness.

^c^ Score range = 0–33, higher scores indicate increasing disability.

^d^ Score range = 0–34, higher scores indicate increasing ageism.

^†^ Based on Fisher's exact test.

### 3.2 HIV‐related characteristics of OALWH

The majority of the OALWH had disclosed their HIV status (95.3%) and were on first‐line cART treatment (90%). The mean (SD) duration of HIV treatment was 11.4 (4.3) years. Nearly, all (98.1%) of the OALWH had a viral load of ≤1000 copies/ml (Table 2).

**Table 2:** HIV‐related clinical and psychosocial characteristics of OALWH, *n =* 257

Characteristic	Mean (SD) or frequency (%)
HIV status disclosure	
Yes	245 (95.3%)
No	12 (4.7%)
Household HIV burden, mean (SD); OM = 5	1.4 (1.6)
cART regimen	
First line	233 (90.7%)
Second line	23 (8.9%)
Third line	1 (0.4%)
cART regimen change/interruption since HIV diagnosis	
Yes	110 (42.8%)
No	147 (57.2%)
Duration on cART (years), mean (SD), OM = 10	11.4 (4.3)
Viral suppression, OM = 45	
Yes	208 (98.1%)
No	4 (1.9%)
Access to HIV care, OM = 4	
Easily accessible	169 (66.8%)
Not easily accessible	84 (33.2%)
Perceived HIV‐stigma score, OM = 1	
Personalized stigma^a^—mean (SD)	5.0 (1.9)
Disclosure concerns^b^—mean (SD)	8.6 (2.0)
Concerns about public attitudes^c^—mean (SD)	7.6 (2.2)
Negative self‐image^d^—mean (SD)	6.4 (2.1)
Overall stigma^e^—mean (SD)	27.5 (5.4)

Abbreviations: cART, combination antiretroviral therapy; OM, observation with missing value; OALWH, older adults living with HIV.

^a^ Score range = 3–12, higher scores indicate greater stigma.

^b^ Score range = 3–12, higher scores indicate greater stigma.

^c^ Score range = 3–12, higher scores indicate greater stigma.

^d^ Score range = 3–12, higher scores indicate greater stigma.

^e^ Score range = 12–44, higher scores indicate greater stigma.

### 3.3 Prevalence estimates for CMDs

The prevalence of mild depressive symptoms was 23.8% among OALWH compared to 18.2% in the comparison group (*p =* 0.16). The prevalence of mild anxiety symptoms was 11.7% among OALWH compared to 7.2% in the comparison group (*p =* 0.12). The prevalence of comorbid mild depressive and anxiety symptoms among OALWH was 10.1% compared to 4.4% in the comparison group (*p =* 0.03) (Table 3).

**Table 3:** Prevalence of common mental disorders in OALWH versus their HIV‐negative peers

	Older adults without HIV, *n =* 181		Older adults living with HIV, *n =* 256		
	Number	Prevalence (95% CI)	Number	Prevalence (95% CI)	*p*‐value
Severity of depressive symptoms					
Mild	28	15.5 (10.5, 21.6)	49	19.1 (14.5, 24.5)	0.3^†^
Moderate	5	2.8 (0.9, 6.3)	8	3.1 (1.4, 6.1)	
Severe	–	–	4	1.6 (0.4, 4.0)	
Positive depressive symptoms screen (cut‐off ≥5)					
Yes	33	18.2 (12.9, 24.6)	61	23.8 (18.7, 29.5)	0.2
Severity of anxiety symptoms					
Mild	13	7.2 (3.9, 12.0)	26	10.2 (6.7, 14.5)	0.1^†^
Moderate	–	–	4	1.6 (0.4, 4.0)	
Positive anxiety symptoms screen (cut‐off ≥5)					
Yes	13	7.2 (3.9, 12.0)	30	11.7 (8.0, 16.3)	0.1
Positive screen for comorbid depressive and anxiety symptoms					
Yes	8	4.4 (1.9, 8.4)	26	10.1 (6.7, 14.5)	0.03

Abbreviation: 95% CI, 95% confidence interval; OALWH, older adults living with HIV.

^†^ Based on Fisher's exact test.

### 3.4 Association between HIV status and CMDs

In univariate and multivariate logistic regression analyses, HIV seropositivity was not significantly associated with depressive or anxiety symptoms (Table 4). However, HIV seropositivity was significantly associated with higher odds of a positive screen for depressive and anxiety symptoms co‐occurrence in univariate but not in the multivariate model.

**Table 4:** Association between HIV status and common mental disorders across the whole sample of older adults

	Positive screen for depressive symptoms		Positive screen for anxiety symptoms		Comorbid depressive and anxiety symptoms	
	Crude analysis	Adjusted analysis	Crude analysis	Adjusted analysis	Crude analysis	Adjusted analysis
Covariate	OR (95% CI)	aOR (95% CI)	OR (95% CI)	aOR (95% CI)	OR (95% CI)	aOR (95% CI)
HIV status						
Seronegative	Ref	Ref	Ref	Ref	Ref	Ref
Seropositive	1.40 (0.87, 2.25)	0.54 (0.27, 1.07)	1.72 (0.87, 3.39)	0.46 (0.18, 1.19)	2.46* (1.09, 5.57)	0.74 (0.25, 2.22)
Sex						
Male		Ref		Ref		Ref
Female		1.68 (0.84, 3.38)		2.49 (0.95, 6.54)		1.93 (0.67, 5.56)
Age (years)						
50–59		Ref		Ref		Ref
60–69		0.89 (0.46, 1.70)		1.78 (0.71, 4.47)		1.72 (0.59, 4.98)
Above 70		0.56 (0.19, 1.69)		3.99* (1.08, 14.71)		3.94 (0.92, 16.94)
Marital status						
Never married		Ref		Ref		Ref
Separated/Divorced/Widowed		0.14* (0.03, 0.69)		0.40 (0.06, 2.75)		0.83 (0.06, 11.27)
Married/cohabiting		0.12* (0.02, 0.62)		0.48 (0.07, 3.53)		1.26 (0.09, 18.17)
Asset Index score		0.77* (0.60, 0.98)		1.06 (0.77, 1.46)		0.91 (0.63, 1.32)
Sexually active						
No		Ref		Ref		Ref
Yes		2.61* (1.24, 5.47)		0.96 (0.38, 2.43)		0.81 (0.28, 2.29)
Functional disability score		1.18** (1.09, 1.28)		1.14* (1.05, 1.24)		1.14* (1.04, 1.24)
Loneliness score		1.12* (1.03, 1.22)		1.16* (1.04, 1.30)		1.12 (0.99, 1.27)
Ageism score		1.10** (1.04, 1.16)		1.16** (1.08, 1.24)		1.16** (1.08, 1.25)
Caring for a sick family member		2.08* (1.08, 4.02)		3.50* (1.32, 9.28)		3.07* (1.03, 9.20)
Chronic fatigue		3.14** (1.46, 6.72)		1.80 (0.69, 4.67)		1.86 (0.66, 5.24)
Sleeping difficulties for the past month						
None		Ref		Ref		Ref
Sometimes		4.62** (2.38, 8.98)		3.64* (1.47, 9.03)		4.69* (1.59, 13.88)
Most of the time/always		10.89** (3.57, 33.24)		4.89* (1.35, 17.63)		7.30* (1.76, 30.27)
Number of the final model		431		431		431
Hosmer–Lemeshow test		*X* ^2^ = 433.90; *p* = 0.25		*X* ^2^ = 307.20; *p* = 0.99		*X* ^2^ = 338.67; *p* = 0.99
Variance explained		35.0%		36.4%		38.8%

Abbreviations: aOR, adjusted odds ratio; CMD, common mental disorder; GAD, generalized anxiety disorder; OR, odds ratio; Ref, reference group.

^*^ *p*‐value < 0.05, ^**^ *p*‐value < 0.001.

### 3.5 Determinants of CMDs in OALWH

Table 5 presents results from logistic regression analyses exploring the determinants of CMDs among OALWH.

**Table 5:** Univariate and multivariable analysis of correlates of common mental disorders among OALWH

	Positive screen for depressive symptoms		Positive screen for anxiety symptoms		Comorbid depressive and anxiety symptoms	
Covariate	Univariate analysis OR (95% CI)	Multivariable analysis aOR (95% CI)	Univariate analysis OR (95% CI)	Multivariable analysis aOR (95% CI)	Univariate analysis OR (95% CI)	Multivariable analysis aOR (95% CI)
Age (years)						
50–59	Ref	Ref	Ref	Ref	Ref	Ref
60–69	0.77 (0.41, 1.44)	0.55 (0.23, 1.32)	1.18 (0.51, 2.72)	2.03 (0.66, 6.20)	1.12 (0.45, 2.76)	0.73 (0.19, 2.84)
≥70	1.35 (0.44, 4.15)	1.00 (0.19, 5.24)	4.19** (1.27, 13.80)	7.43** (1.25, 44.36)	4.55** (1.37, 15.09)	4.76 (0.70, 32.55)
Sex						
Male	Ref	Ref	Ref	Ref	Ref	Ref
Female	1.34 (0.73, 2.45)	1.36 (0.58, 3.19)	1.79 (0.76, 4.19)	2.17 (0.58, 8.08)	1.73 (0.70, 4.29)	2.59 (0.64, 10.45)
Marital status						
Never married	Ref	–	–	–	–	–
Separated/Divorced/Widowed	0.19** (0.04, 0.82)	–	–	–	–	–
Married/cohabiting	0.16** (0.04, 0.73)	–	–	–	–	–
Education level						
None	Ref	–	Ref	–	Ref	–
Primary	0.80 (0.41, 1.57)	–	0.51* (0.22, 1.19)	–	0.52* (0.22, 1.25)	–
Secondary	0.78 (0.34, 1.80)	–	0.38* (0.11, 1.24)	–	0.20** (0.04, 0.96)	–
Tertiary	0.16* (0.02, 1.28)	–	0.28 (0.03, 2.30)	–	0.31 (0.04, 2.59)	–
Monthly household income (Ksh)						
≤10,000	Ref	–	Ref	–		
Above 10,000	0.33*** (0.12, 0.86)	–	0.13* (0.02, 1.02)	–	0.16* (0.02, 1.22)	–
Living arrangements						
Multiple generational families	–	–	–	–	Ref	–
Single generational families	–	–	–	–	2.11* (0.77, 5.78)	–
Alone	–	–	–	–	1.05 (0.29, 3.82)	–
Number of dependents, mean (SD)	–	–	0.87* (0.74, 1.04)	–	–	–
Caring for a sick family member						
No	Ref	–	–	–	–	–
Yes	0.63* (0.33, 1.16)	–	–	–	–	–
Food insecurity (lack of food in the past week)						
Never	Ref		Ref	–	Ref	–
Sometimes	2.92*** (1.55, 5.49)	–	4.21*** (1.75, 10.14)	–	5.95*** (2.18, 16.20)	–
Most of the times/always	2.9** (1.11, 7.62)	–	6.21** (1.96, 19.70)	–	9.56*** (2.76, 33.15)	–
Loneliness score, mean (SD)	1.21*** (1.12, 1.32)		1.29*** (1.16, 1.43)	1.16** (1.01, 1.34)	1.28*** (1.15, 1.42)	
Functional disability score, mean (SD)	1.25*** (1.15, 1.35)	1.15** (1.04, 1.28)	1.19*** (1.10, 1.28)	1.10** (1.01, 1.20)	1.18*** (1.10, 1.27)	1.12** (1.03, 1.22)
Ageism score, mean (SD)	1.14*** (1.08, 1.19)	1.10** (1.04, 1.17)	1.17*** (1.10, 1.24)	1.14*** (1.06, 1.23)	1.17*** (1.11, 1.24)	1.23*** (1.13, 1.33)
Hours spent in sedentary behaviours in a day, mean (SD)	–	–	–	–	1.13* (1.00, 1.28)	–
Sexually active						
No	–	–	Ref	–	Ref	–
Yes	–	–	0.44* (0.18, 1.06)	–	0.44* (0.17, 1.15)	–
Sleeping difficulties in the past month						
None	Ref	Ref	Ref	Ref	Ref	–
Sometimes	6.59*** (3.18, 13.66)	3.51** (1.38, 8.89)	10.17*** (3.29, 31.47)	6.41** (1.73, 23.83)	11.75*** (3.28, 42.0)	–
Most of the times/always	25.60*** (9.00, 72.99)	8.82*** (2.33, 33.37)	18.25*** (4.94, 67.40)	6.18** (1.26, 30.22)	20.31*** (4.80, 85.96)	–
Chronic fatigue						
No	Ref	Ref	Ref	–	Ref	–
Yes	7.90*** (3.66, 17.02)	5.41** (1.78, 16.38)	2.68** (1.09, 6.62)	–	3.36** (1.33, 8.46)	–
Number of medications participants are currently using, mean (SD)	–	–	–	–	1.24* (0.94, 1.64)	–
HIV status disclosure						
Yes	Ref	–	–	–	–	–
No	2.40* (0.73, 7.85)	–	–	–	–	–
Household HIV burden, mean (SD)	1.30** (1.09, 1.55)	1.37** (1.08, 1.74)	1.20* (0.98, 1.47)	–	1.21* (0.98, 1.49)	–
cART regimen change/interruption since HIV diagnosis						
No	–	–	Ref	–	Ref	–
Yes	–	–	2.13* (0.97, 4.68)	–	1.88* (0.82, 4.34)	–
Access to HIV care						
Not easily accessible	Ref	Ref	Ref	–	Ref	Ref
Easily accessible	0.47** (0.26, 0.85)	0.35** (0.15, 0.83)	0.36** (0.16, 0.78)	–	0.29** (0.12, 0.68)	0.16** (0.04, 0.60)
Perceived HIV‐stigma score, mean (SD)						
Personalized stigma	1.21** (1.04, 1.41)	1.27** (1.02, 1.59)	1.16* (0.96, 1.42)	–	1.25** (1.02, 1.54)	–
Disclosure concerns	–	–	–	–	–	–
Concerns about public attitudes	1.23** (1.06, 1.42)	–	1.29** (1.06, 1.57)	–	1.23** (1.00, 1.51)	–
Negative self‐image	1.22** (1.06, 1.40)	–	1.20** (1.00, 1.44)	–	1.36** (1.12, 1.66)	–
Overall stigma	1.11*** (1.05, 1.18)	–	1.10** (1.02, 1.20)	–	1.13** (1.04, 1.23)	–
*n* for the final model		251		252		245
Variance explained		41.7%		41.0%		42.0%
Hosmer–Lemeshow test		*X* ^2^ = 241.96; *p*‐value = 0.35		*X* ^2^ = 210.85; *p*‐value = 0.79		*X* ^2^ = 176.09; *p*‐value = 0.99
cvMean AUC (95% CI)		0.91 (0.87, 0.96)		0.88 (0.82, 0.95)		0.92 (0.89, 0.96)

Note: Only a priori variables (age and sex), as well as those with *p*‐value < 0.15 in the univariate analysis or multivariable *p* < 0.05, are presented here. Ten independent variables were fitted for the multivariable model on depressive symptoms, seven variables for both the anxiety symptoms and CMD comorbidity.

Abbreviations: aOR, adjusted odds ratio; cvMean AUC, cross‐validated mean area under the curve for the final multivariable model; OR, odds ratio; Ref, reference group; OALWH, older adults living with HIV.

^*^ *p* value < 0.15, ^**^ *p* value < 0.05, ^***^ *p* value < 0.01.

#### 3.5.1 Depressive symptoms

In the multivariable logistic regression model, factors significantly associated with higher odds of depressive symptoms among OALWH were functional disability, ageism, sleeping difficulties, chronic fatigue, increasing household HIV burden and perceived HIV‐related stigma. Easier access to HIV care was significantly associated with lower odds of depressive symptoms.

#### 3.5.2 Anxiety symptoms

In the multivariable analyses, age ≥70, perceived loneliness, functional disability, ageism and sleeping difficulties were significantly associated with higher odds of anxiety symptoms among OALWH.

#### 3.5.3 Comorbid depressive and anxiety symptoms

In the multivariable analyses, perceived functional disability and ageism were significantly associated with higher odds of comorbid depressive and anxiety symptoms. Easier access to HIV care was significantly associated with lower odds of comorbid depressive and anxiety symptoms.

### 3.6 Determinants of CMDs in HIV‐negative older adults

The current work largely focused on OALWH. As such, we only provide a summary of the factors associated with CMDs among the HIV‐negative older adults using the available data, particularly focusing on mild depressive symptoms whose prevalence was relatively high in the current sample, that is >10%. In multivariable analyses (Table S1), sleeping difficulties, perceived loneliness and increasing medication burden were significantly associated with higher odds of a positive screen for depressive symptoms among HIV‐negative older adults. Greater sedentary behaviour and higher monthly household income were significantly associated with lower odds of depressive symptoms.

## 4 DISCUSSION

To our knowledge, this is the first study in Kenya and among the first reports from SSA investigating CMDs among OALWH compared to their HIV‐negative peers. Our study found a relatively high burden of mild depressive and anxiety symptoms; however, HIV status was not independently associated with these symptoms. The correlates of CMDs in our study were predominantly psychosocial factors, many of which are potentially modifiable, thus highlighting the need to address the psychosocial needs of these adults alongside their biomedical needs.

Our finding of no significant differences in the prevalence of CMDs between OALWH and their HIV‐negative peers is dissimilar to other emerging reports in SSA. In rural Uganda, OALWH on cART had a significantly lower prevalence of probable depression than their HIV‐negative peers [36], similar to what was reported in rural South Africa [35]. Our finding also offers an interesting contrast to the predominant findings in HICs showing that OALWH present with worse CMDs than their HIV‐negative peers [24]. The observed variation could reflect contextual differences across settings, for example healthcare systems, informal support systems and mental health resources that are likely to alter the risk profile of these adults. HIV status was not found to be an independent predictor of CMDs in our study. This observation is consistent with previous findings from South Africa [49] and Uganda [19] but contrasts with common research findings in HICs [24]. Our study may be part of an emerging body of evidence in SSA showing that the psychological health of OALWH is not worse than among those without HIV.

HIV‐related stigma was significantly associated with higher odds of depressive symptoms among OALWH in our study. These findings are consistent with previous findings in the literature [12, 16, 50] and provide additional evidence of the critical role of addressing intersecting stigma in improving the mental wellbeing of OALWH. Relatedly, ageism was also significantly associated with higher odds of depressive symptoms, anxiety symptoms and their co‐occurrence among OALWH. This finding is consistent with previous research conducted outside SSA [51]. Ageism could have an adverse impact on the mental health of OALWH through psychological, behavioural and physiological pathways [52]. Interventions addressing both ageist and HIV‐stigmatizing attitudes at the community level will potentially improve the mental health of OALWH.

Increasing household HIV burden was significantly associated with higher odds of depressive symptoms among OALWH in our study. This may be related to the high caregiving burden often experienced by OALWH caring for HIV‐positive children. This observation concurs with previous findings in the study setting [53].

Loneliness is common among older adults in general [54]. In this study, higher perceived loneliness was significantly associated with elevated odds of anxiety symptoms among OALWH and depressive symptoms among HIV‐negative older adults. Our finding is consistent with previous studies from HICs [12, 55]. Theoretical models suggest that loneliness has cognitive, biological and social consequences that could potentially heighten the risk of subsequent CMDs [56]. Higher perceived functional disability was also strongly associated with higher odds of depressive symptoms, anxiety symptoms and their co‐occurrence among OALWH in the current study, consistent with previous findings [19, 57, 58]. Since functional disability occurs frequently among OALWH, there is a need for early identification to help preserve functional independence.

Sleep disturbance is a prominent symptom in people with CMDs, especially depression, and was formerly regarded as a main secondary indicator of depression [59]. Nonetheless, multiple prospective studies have identified insomnia as an independent risk indicator for emerging or recurrent depression, suggesting that sleep problems are not necessarily secondary effects of CMDs but a predictive prodromal symptom [60, 61]. In this study, persistent sleep problems were significantly associated with higher odds of depressive symptoms, anxiety symptoms and their co‐occurrence among OALWH and higher odds of depressive symptoms among HIV‐negative older adults. This finding is consistent with previous findings [59, 62, 63]. A combination of pharmacological and non‐pharmacological interventions for sleep disturbances may effectively reduce and possibly prevent CMDs [64].

Chronic fatigue was also significantly associated with increased odds of depressive symptoms among OALWH in our study, similar to what has been reported elsewhere [65, 66]. Fatigue is a vital indicator of ageing‐related declines in health and functioning [67]. Fatigue management strategies, such as adequate rest and sleep, are likely to improve the mental health of OALWH. Among HIV‐negative older adults, an increased medication burden was also significantly associated with higher odds of depressive symptoms, consistent with previous findings [68]. While the exact mechanism for this association is unknown, we know that the use of medication increases as the number of medical conditions rises. Multiple medications, which are easily detected by clinicians, can provide an important clue to healthcare providers to further investigate depression in their clients.

Among socio‐demographic factors, old age (≥70 years) was significantly associated with higher odds of anxiety symptoms in OALWH, while higher monthly household income was significantly associated with lower depressive symptoms among HIV‐negative older adults. Mixed findings have been reported on these factors previously [69].

Easier access to HIV care was the only protective indicator for CMDs in OALWH in the current study. Given that many OALWH in Kenya face unique challenges with seeking HIV care services [70], programmes aimed at strengthening HIV care access or financial support have the potential to improve OALWHs’ mental wellbeing. Further decentralizing HIV care into the community possibly utilizing community health workers may also be beneficial.

Among HIV‐negative older adults, an increasing number of hours on sedentary activities was significantly associated with lower odds of depressive symptoms. More studies are needed to better understand the mechanism involved. Emerging data suggest that passive sedentary behaviours, for example television watching, increase the risk of depression, while mentally active sedentary behaviours, for example reading, may be protective against depression [71].

Most of our data (about 84%) were collected after the onset of the COVID‐19 pandemic. Some studies have reported elevated levels of loneliness, depression, anxiety and insomnia among older adults following the outbreak of COVID‐19 [72, 73, 74], while others have reported no changes before and during the pandemic despite increased loneliness during the pandemic [75]. Other studies have shown that younger populations have had higher rates of CMDs compared to older adults [76, 77, 78, 79]. While it is possible that the emergence of the pandemic may have created an environment where the determinants of poor mental health could have been exacerbated, our study found low prevalences of CMDs, similar to previous research, suggesting higher resilience to the mental health effects of COVID‐19 [80]. The long‐term impacts of the pandemic remain unclear, especially in SSA where data on older adults’ mental health are very scarce. More studies are needed to elucidate these findings.

The strengths of the current study include the focus on a neglected but rapidly growing population of OALWH, the use of a comparison group and sufficient sample size. Nonetheless, the cross‐sectional nature of the study precludes any conclusion on causality. We recruited our OALWH from public HIV clinics, as such, our findings may not be readily generalizable to OALWH who may be out of care or attending private or urban HIV clinics or recruited from the community. We also utilized self‐report screening measures which could be subject to reporting bias. Relatedly, the mental health screening measures do not give a clinical diagnosis of the studied CMDs, hence, we only report the symptomatology of these conditions.

Despite the outlined limitations, this study has important implications for the care of older adults in our setting. We observed substantial levels of mild depressive and anxiety symptoms in both OALWH and their HIV‐negative peers, highlighting the need for culturally appropriate mental health interventions in these older adults, regardless of their HIV status. Routine screening for CMDs should be strengthened to identify those at risk. Risk indicators for depressive symptoms, anxiety symptoms and their co‐occurrence in this study were predominantly psychosocial factors. Unfortunately, there is a paucity of research on psychosocial interventions among OALWH [81] and those in the general population [82], especially in SSA. Our findings highlight the need to strengthen the evidence base for interventions for CMDs among older adults in low‐resource settings like Kenya.

## 5 CONCLUSIONS

Ambulatory, out‐patient OALWH and their HIV‐negative peers from the community have similar levels of mild depressive and anxiety symptoms. Additionally, living with HIV is not predictive of CMDs in this setting. Our study provides an initial understanding of the determinants of CMDs from a low‐resource setting. Modifiable risk factors, such as ageism, HIV‐related stigma, loneliness, functional disability and sleeping difficulties, represent a target for preventive interventions through psychosocial interventions at the family, community and clinical levels.

## COMPETING INTERESTS

The authors have no competing interests to disclose.

## AUTHORS’ CONTRIBUTIONS

PNM, CRN and AA conceptualized the study. PNM, CRN, RGW and AA designed the study. PNM and CN programmed the study questions on tablets and managed project data for the entire study period. PNM analysed the data. PNM, CN, RGW, CRN and AA contributed to the interpretation of the data. PNM wrote the first draft of the manuscript and all the authors reviewed the subsequent versions and approved the final draft for submission.

## FUNDING

This work was funded by the Wellcome Trust International Master's Fellowship to PNM (grant number 208283/Z/17/Z). Further funding supporting this work was from (1) the Medical Research Council (grant number MR/M025454/1) to AA. This award is jointly funded by the UK Medical Research Council (MRC) and the UK Department for International Development (DFID) under MRC/DFID concordant agreement and is also part of the EDCTP2 programme supported by the European Union; (2) DELTAS Africa Initiative [DEL‐15‐003]. The DELTAS Africa Initiative is an independent funding scheme of the African Academy of Sciences (AAS) ’s Alliance for Accelerating Excellence in Science in Africa (AESA) and supported by the New Partnership for Africa's Development Planning and Coordinating Agency (NEPAD Agency) with funding from the Wellcome Trust [107769/Z/10/Z] and the UK government. The funders did not have a role in the design and conduct of the study or interpretation of study findings.

## DISCLAIMER

The views expressed in this publication are those of the author(s) and not necessarily those of AAS, NEPAD Agency, Wellcome Trust or the UK government. For the purpose of Open Access, the author has applied a CC‐BY public copyright license to any accepted manuscript version arising from this submission.

## Supporting information


**Table S1**. Univariate and multivariable analysis of the correlates of depressive symptoms among HIV‐negative older adults.

## Data Availability

Application for data access can be made through the Data Governance Committee of the KEMRI Wellcome Trust Research Programme who will review the application and advise as appropriate ensuring that uses are compatible with the consent obtained from participants for data collection. Requests can be sent to the coordinator of the Data Governance Committee using the following email dgc@kemri-wellcome.org.
