# Supplementary material for: Prevalence and factors associated with mild depressive and anxiety symptoms in older adults living with HIV from the Kenyan coast
Source: J Int AIDS Soc. 2022 Sep 29;25(Suppl 4):e25977. doi: 10.1002/jia2.25977 (PMC9522642; doi:10.1002/jia2.25977)
Supplement: Supplementary file 1 — Table S1. Univariate and multivariable analysis of the correlates of depressive symptoms among HIV‐negative older adults. [file JIA2-25-e25977-s001.docx]

**Supplementary Table 1.** Univariate and multivariable analysis of the correlates of depressive symptoms among HIV-negative older adults

| **Covariate** | Univariate analysis OR (95% CI) | Multivariable analysis aOR (95% CI) |
| --- | --- | --- |
| **Age (years)** |  |  |
| *50 – 59* | Ref | Ref |
| *60 – 69* | 1.21 (0.55, 2.69) | 1.18 (0.42, 3.27) |
| *≥70* | 0.63 (0.17, 2.37) | 0.19 (0.02, 1.51) |
| **Sex** |  |  |
| *Male* | Ref | Ref |
| *Female* | 2.00***** (0.91, 4.42) | 1.00 (0.34, 2.96) |
| **Monthly household income (Ksh)** |  |  |
| *≤10,000* | Ref | Ref |
| *Above 10,000* | 0.31******* (0.14, 0.68) | 0.33****** (0.11, 0.94) |
| **Number of dependents, mean (SD)** | 1.11***** (0.96, 1.29) | – |
| **Caring for a sick family member** |  |  |
| *No* | Ref | – |
| *Yes* | 1.93***** (0.86, 4.33) | – |
| **Food insecurity (lack of food in the past week)** |  |  |
| *Never* | Ref | – |
| *Sometimes* | 2.42****** (1.08, 5.42) | – |
| *Most of the times/always* | 2.97 (0.26, 34.43) | – |
| **Loneliness score, mean (SD)** | 1.22******* (1.09, 1.37) | 1.23******(1.05, 1.44) |
| **Functional disability score, mean (SD)** | 1.13****** (1.01, 1.26) | – |
| **Ageism score, mean (SD)** | 1.10****** (1.02, 1.19) | – |
| **Hours spent in sedentary behaviours in a day, mean (SD)** | 0.69******* (0.56, 0.86) | 0.72****** (0.55, 0.94) |
| **Sleeping difficulties in the past month** |  |  |
| *No* | Ref | Ref |
| *Yes* | 5.87******* (2.62, 13.14) | 6.91******* (2.38, 20.08) |
| **Chronic fatigue** |  |  |
| *No* | Ref | – |
| *Yes* | 3.32****** (1.25, 8.84) | – |
| **Number of medications participants are currently using, mean (SD)** | 1.48****** (1.02, 2.14) | 1.37****** (1.01, 1.89) |
| **n for the final model** | – | 178 |
| **Variance explained** | – | 34.5% |
| **Hosmer-Lemeshow test** | – | X^2^ = 200.21; p-value = 0.22 |
| **cvMean AUC (95% CI)** | – | 0.83 (0.79, 0.88) |
| Only a priori variables (age, sex), as well as those with p-value < 0.15 in the univariate analysis or multivariable *p* < 0.05 are presented here.  *OR* odds ratio, *aOR* adjusted odds ratio, *Ref* reference group, *cvMean AUC* cross-validated mean area under the curve for the final multivariable model  ***** - *p* value< 0.15, ****** - *p* value < 0.05, ******* - *p* value < 0.01 | | |
